# Altered Fruit and Seed Development of Transgenic Rapeseed (*Brassica napus*) Over-Expressing MicroRNA394

**DOI:** 10.1371/journal.pone.0125427

**Published:** 2015-05-15

**Authors:** Jian Bo Song, Xia Xia Shu, Qi Shen, Bo Wen Li, Jun Song, Zhi Min Yang

**Affiliations:** 1 Department of Biochemistry and Molecular Biology, College of Life Science, Nanjing Agricultural University, Nanjing, China; 2 The Rapeseed Institute of Guizhou Province, Gui Yang, China; 3 Department of Biochemistry and Molecular Biology, College of Life Science, Jiangxi Agricultural University, Nanchang, China; NSW Department of Primary Industries, AUSTRALIA

## Abstract

Fruit and seed development in plants is a complex biological process mainly involved in input and biosynthesis of many storage compounds such as proteins and oils. Although the basic biochemical pathways for production of the storage metabolites in plants are well characterized, their regulatory mechanisms are not fully understood. In this study, we functionally identified rapeseed (*Brassica napus*) miR394 with its target gene *Brassica napus LEAF CURLING RESPONSIVENESS* (*BnLCR*) to dissect a role of miR394 during the fruit and seed development. Transgenic rapeseed plants over-expressing miR394 under the control of the cauliflower mosaic virus 35S promoter were generated. miR394 over-expression plants exhibited a delayed flowering time and enlarged size of plants, leaf blade, pods and seed body, but developed seeds with higher contents of protein and glucosinolates (GLS) and lower levels of oil accumulation as compared to wild-type. Over-expression of miR394 altered the fatty acid (FA) composition by increasing several FA species such as C16:0 and C18:0 and unsaturated species of C20:1 and C22:1 but lowering C18:3. This change was accompanied by induction of genes coding for transcription factors of FA synthesis including *LEAFY COTYLEDON1* (*BnLEC1*), *BnLEC2*, and *FUSCA3* (*FUS3*). Because the phytohormone auxin plays a crucial role in fruit development and seed patterning, the DR5-GUS reporter was used for monitoring the auxin response in Arabidopsis siliques and demonstrated that the DR5 gene was strongly expressed. These results suggest that BnmiR394 is involved in rapeseed fruit and seed development.

## Introduction

Rapeseed (oilseed rape, *Brassica napus*) is one of the major crops with great economical importance for its seed oil for human nutrition and protein for animal foodstuffs. For Brassicaceae family species like Arabidopsis and *Brassica napus*, a fruit, morphologically defined as a silique (or pod), is a specialized organ developed from gynoecium after fertilization of ovules [1, 2]. The fruit development is physiologically coupled with embryo development and seed maturation. An essential metabolic function of maturing seeds is the deposition of storage compounds such as lipid of triacylglycerols (TAGs) and proteins. Seeds of *Brassica napus* may accumulate storage lipids up to 50% or more based on their dry weight. As an excellent model system, the Brassica species seed development has become a major focus of plant research on the genetic control of seed filling with storage compounds [3].

A variety of genes involved in fruit and seed development have been identified recently from Brassica species. For example, the MADS-box gene *FRUITFULL* (*FUL*) was found to regulate valve development [4]. The *FUL1* loss of function during Arabidopsis fruit development resulted in the failure of valve cells to elongate and differentiate. With respect to oilseed rape, an interest is placed on the manipulation of lipid levels through the genes involved in FA biosynthesis and triacylglycerol (TAG) assembly [5]. Genes controlling TAG biosynthesis and other relevant components have been well characterized, but the regulatory mechanism of the pathways is poorly understood. Recently, a group of microRNAs (miRNAs) have been found to involve embryo and seed development in plants [6–8]. miRNAs are a class of short (~21 nt) endogenous non-coding small RNAs that base pair sequences complementary with specific target genes to repress their translation or induce their degradation [9]. With genome-wide profiling of small RNAs from rapeseed crops, a large number of miRNAs involved in plant growth, development and environmental stresses have been identified [10–17]. However, only few of miRNAs were functionally characterized as of today. Recently, miR397 from rice (*Oryza sativa*) has been identified to target an *Oryza sativa* laccase-like protein (*OsLAC*) gene, which involves plant sensitivity to brassinosteroids, grain size and panicle branching [18]. Also, ectopic expression of apple miR156h in Arabidopsis resulted in an extended juvenile phase, increased numbers of leaves, short siliques and the partial abortion of seeds [19]. We recently identified Arabidopsis miR394 and its target gene *LCR* (*LEAF CURLING RESPONSIVENESS*) and showed that both miR394 and LCR can coordinately regulate leaf morphology [20]. Furthermore, miR394 has been found to involve stem cell identity in Arabidopsis [21]. In this report, we show that *Brassica napus* miR394 with its target gene *BnLCR* is involved in rapeseed fruit and seed development. miR394 over-expression resulted in morphological changes in fruits and seeds. The storage oil contents and composition as well as protein and GLS were altered in the transgenic rapeseed over-expressing miR394. We further provided evidence that elevated *BnLCR* transcripts could also lead to defective phenotypes in fruit and seed developments. These results indicate that a proper miR394 level is necessary for the normal development of fruit and formation of seed of *Brassica napus*.

## Materials and Methods

### Plant materials and cultivation

Seeds of rapeseed (*Brassica napus* L. Youyan No. 9) were surface-sterilized and uniformly germinating seeds were planted in pots (60 L) with soil (Yellow-brown soil, Eutric gleysols) at Experimental Station of Nanjing Agricultural University. The soil used was air-dried, gently crumbled and passed through a 2-mm sieve, with its basic properties summarized in Table A in S1 File. For Arabidopsis (ecotype, Col-0) plants, sterilized seeds were grown under hydroponic conditions and vernalized in darkness at 4°C for 2 days before the plate was transferred to a growth chamber under the conditions of 16/8 h (day/night), 200 μE m^-2^ s^-1^ photosynthetically active radiation. After that, seedlings (seven day-old) were transferred to soil and placed in the growth chamber under the same condition.

### Plant transformation

pCAMBIA1304 was used as the plant expression vector with or without CaMV35S as a promoter and NOS terminator as transcriptional termination sequences [22]. The target genes were PCR-amplified using primers with restriction enzyme sites at the 5'-end of forward and reverse primers, respectively. PCR amplified component sequences were first cloned to a T/A vector (pMD19, Takara), sequenced and digested. The digested segments were cloned into pCAMBIA1304 and the cloning was confirmed by sequencing and restriction analysis. The validated clones were transformed into *Agrobacterium tumefaciens* strain LBA4404 following the standard freeze thaw method of transformation. We also constructed an antisense LCR mRNA vector to examine the effect of *LCR* knock-down on fruit or seed development. All vectors containing corresponding genes were transferred into *Brassic napus* (Youyan No. 9) and Arabidopsis via *Agrobacterium*-mediated transformation. In this study, all transgenic plants were used in the form of T3 generation.

### GUS assay

Construction of miR394, LCR and DR5 reporter vectors and histochemical detection of GUS activity were performed based on the previous method [19, 23]. Briefly, the chemical regent 5-bromo-4-chloro-3-indolyl β-D-glucuronic acid (X-Gluc) was used as a substrate. Leaf tissue was placed in X-Gluc solution [750 mg/mL X-Gluc, 100 mM NaPO4, pH 7, 3 mM K_3_F_3_ (CN)_6_, 10 mM EDTA, and 0.1% Nonidet P-40] under a vacuum for 10 min at room temperature, then incubated overnight at 37°C.

### Quantitative RT-PCR analysis

Total RNA from plant tissues was isolated and 1.0 μg RNA was used as templates for cDNA synthesis. Quantitative RT-PCR (qRT-PCR) was conducted on CFX96 Real-Time PCR Detection System (Bio-Rad). Amplification reaction was performed in a 25 μL mixture containing 5 ng template, 12.5μL SYBR-Green PCR Mastermix (Toyoba, Japan) and 10 pmol primers. The temperature profile was 98°C for 30s, followed by 40 cycles at 98°C for 2s, 60°C for 5s and melt curve at 65°C 5s. Data were analyzed using CFX Data Analysis Manager Software. The relative expression level was normalized to that of the *Actin*, which was used as the internal control, with the 2^-△CT^ method representing the relative quantification of gene expression [22]. The primers used for qRT-PCR are presented in Table B in S1 File.

### Analysis of oil, proteins and GLS contents

Total oil, protein, and GLS contents of *Brassica napus* seeds were analyzed using a Foss NIRSystems 5000 near-infrared reflectance spectroscopy (Foss NIRSystems Inc.). Approximate 3.5 g dried seeds were scanned in a 36 mm inner-diameter ring cup. The routine analytical process was run according to WinISI III manual instructions (Foss-tecator Infrasoft International LLC) [24].

The oil content was analyzed further by Soxhlet method using FOSS Fat Extraction System 2500 [25]. Briefly, rapeseed seeds were ground with FOSS sample grinding apparatus 1093. The seed powder was dried at 105°C for two hours and cooled to the room temperature in desiccator. The sample with 0.5 g accurately weighed and quantified for oil content. The sample was transferred into a Soxhlet tube and extracted with n-hexane as extract solvent. Extracting, solvent washing, solvent recovering and drying were performed respectively for 20, 40, 10 and 10 min based on the normalization procedure. The oil content (%) was calculated by the formula (added solvent weigh/sample weigh)×100%. For each sample, triplicates were performed and mean values of the triplicates were used to calculate the oil content. Analysis of fatty acid species was preformed using gas chromatography with the procedure documented.

### Fatty acid composition analysis

The standard fatty acid methyl ester (99% purity) was purchased from Sangon Biotech [26]. The rapeseed seeds (0.5 g) were ground. Lipids were extracted with 5 mL solvent composed of diethyl ether / hexane petroleum ether (1:1, v/v), and fatty acid components of the total glycerolipids were converted into 3 mL methyl esters by transesterification with 0.5 mol L^-1^ NaOH for 30 min. One μL lipid extracted solvent was analyzed by gas chromatograph using Agilent 6890 N. The initial column temperature was 150°C hold 3 min, increasing at 5°C min^-1^ to 220°C, and then hold for 10 min. The peaks corresponding to each fatty acid species were recorded and identified by their characteristic retention times.

### Statistical analysis

All experiments in the study were independently performed in triplicate. Each result shown in the figures was the mean of at least three replicated treatments and each treatment contained at least 30 plants. Unless indicated, the equal amount of mixed transgenic line seeds was used and samples for analysis were randomly selected from all transgenic lines. The significant differences between treatments were statistically evaluated by standard deviation and one-way analysis of variance (ANOVA). The data between differently treated groups were compared statistically by ANOVA, followed by the least significant difference (LSD) test if the ANOVA result was significant at *P*<0.05.

## Results

### Analysis of miR394 and its target gene in *Brassica napus*

In Arabidopsis, miR394 family has two members, miR394a and miR394b with a predicted target gene coding for an F-box protein [27]. The *Brassica napus* miR394 (BnmiR394) was isolated from one of our small RNA libraries prepared from the rapeseed seedlings [12]. By the sequence of mature miR394 against the publicly available databases (NCBI), an ortholog target of miR394 in *Brassica napus* was predicted. Using the rapid amplification of cDNA ends (RACE) approach, a full-length cDNA for the *Brassica napus* target of miR394 was isolated. Therefore, it was assigned as *BnLCR*. The miR394-guided cleavage of *BnLCR* has been confirmed by RACE (Figure A in S1 File) and further by our degradome analysis [16]. The CDS contains a 1404 bp long open reading frame (ORF) coding for a deduced protein of 467 amino acid residues (Figure B in S1 File), whose number is exactly the same as AtLCR from Arabidopsis [20]. Alignment of the deduced amino acid sequence of BnLCR revealed common features, such as an F-box domain at the N-terminal region with 50-amino acids. Phylogenetic analysis showed that BnLCR is grouped together with orthologs from many other plant species (Figure C in S1 File). Isolation of *Brassica napus* DNA sequence corresponding to the *BnLCR* cDNA revealed a gene with the length of 2013 bp containing a 609 bp intron (Figure D in S1 File). These results indicate that both Arabidopsis and *Brassica napus* have the identical LCR genes.

**Fig 1:**
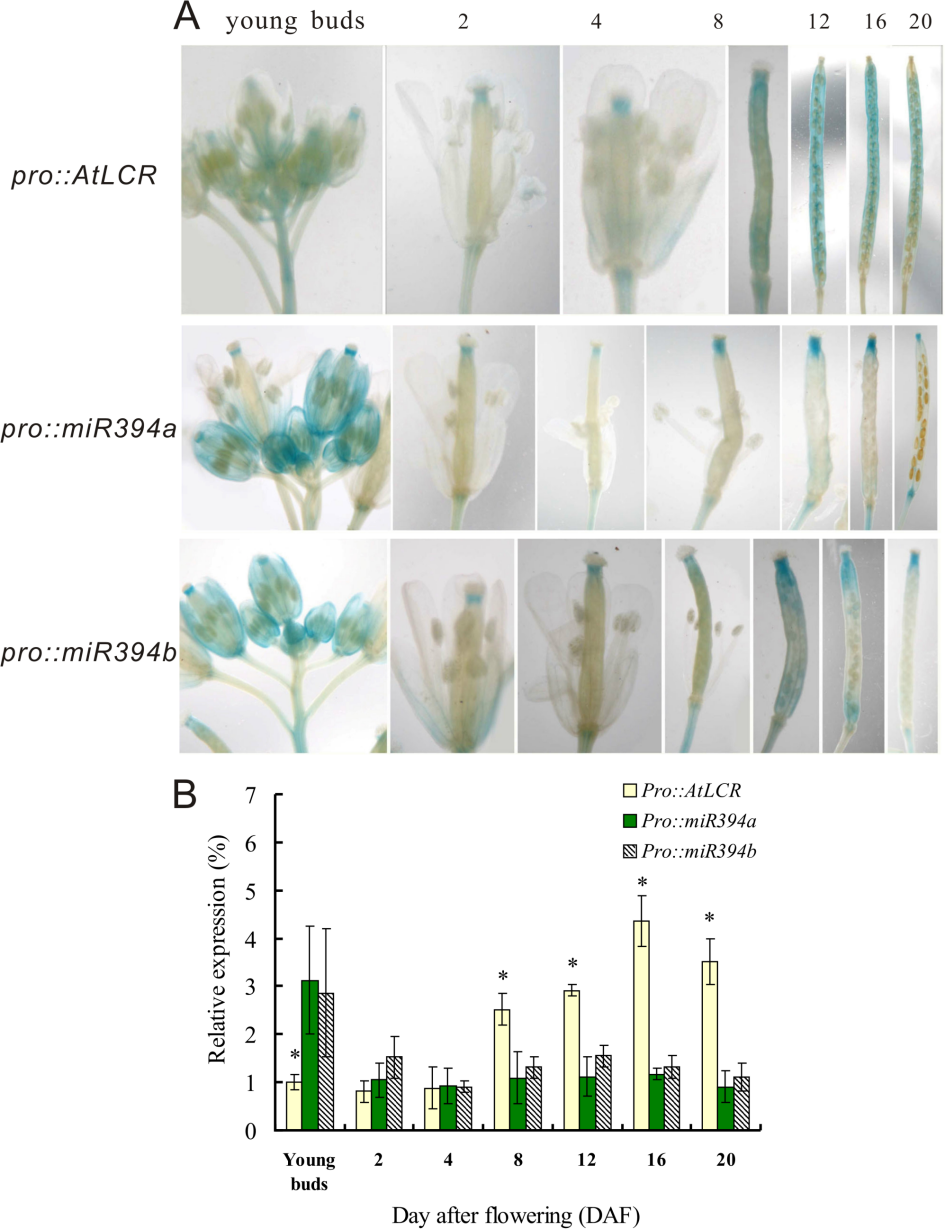
Promoter activity of miR394 and *LCR* in Arabidopsis flowers and developing siliques. A: GUS staining of the transgenic Arabidopsis carrying *pLCR*::*GUS* (*Pro*::*AtLCR*), *pMIR394a*::*GUS* (*Pro*::*miR394a*) and *pMIR394b*::*GUS* (*Pro*::*miR394b*). GUS staining in flowers or siliques was identified with days after flowering (DAF). B: The relative expression of GUS in the transgenic Arabidopsis plants. The intensity was quantified using Adobe Photoshop CS software. Vertical bars represent SD of the mean of treatments (*n* = 3). Asterisks indicate that mean values are significantly different between the GUS intensity of *Pro*::*AtLCR* and *Pro*::*miR394a* or *Pro*::*miR394b* (*P*<0.05).

To show the expression pattern of *Brassica napus* miR394 and its target *LCR* during the fruit and seed development, we developed transgenic Arabidopsis with GUS reporter genes fused to miR394a/b and LCR promoters, and monitored the GUS staining in flowers and fruits. The high GUS activity of *pMIR394a*::*GUS* and *pMIR394b*::*GUS* lines was detected at the early stage of young buds (Fig 1). But two days after flowering (DAF), the GUS activity attenuated but remained a low level throughout 2 to 20 DAF. In contrast, the GUS staining of *pLCR*::*GUS* plants was moderate at the early stage but became strong with the seed development. The highest GUS staining was detected at the late stages (16–20 DAF), when the strong GUS staining was observed nearly full of the siliques.

### miR394 over-expression regulated fruit and seed development of *Brassica napus*

**Fig 2:**
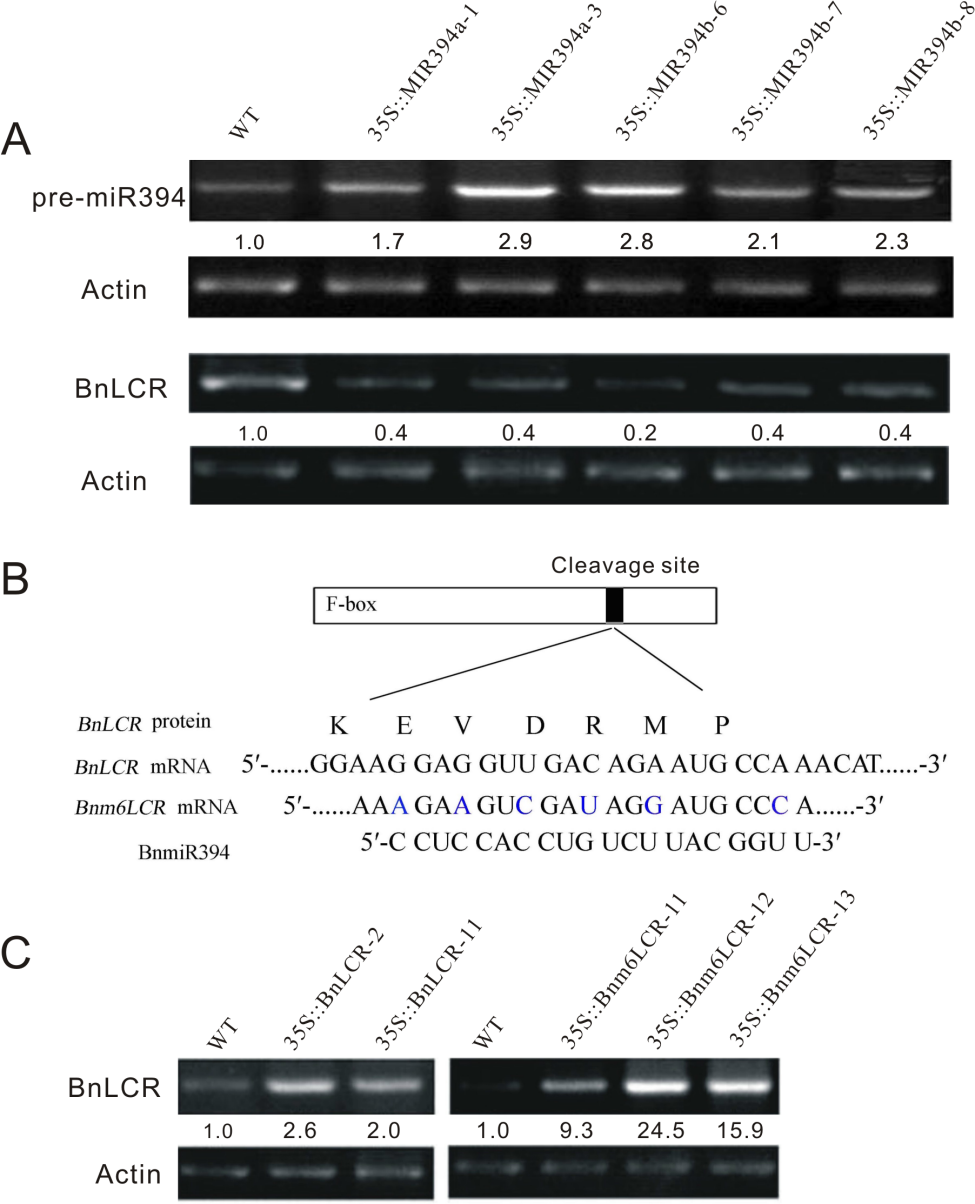
Expression of *Brassica napus* precursor miR394a/b and its target *BnLCR* in wild type (WT) and the transgenic plants. Total RNA from two week-old seedlings was extracted and transcripts were analyzed. A: transcripts of pre-miR394a/b and *BnLCR* were analyzed using semi-quantitative RT-PCR. B: Base paring of miR394 with the corresponding complementary site of *BnLCR* and a miR394-resistant form of *BnLCR* (*Bnm6LCR*). C: Transcripts of *BnLCR* in miR394-resistant (*35S*::*Bnm6LCR*) and miR394 non-resistant (*35S*::*BnLCR*) transgenic lines. Actin was used for cDNA normalization. The number below the band indicates relative abundance of the corresponding gene with respect to the loading controls Actin. or *35S*:*m5LCR* plants and WT (*p* < 0.05).

To investigate the biological function of miR394 during the rapeseed fruit and seed development, transgenic rapeseed plants over-expressing miR394a/b were generated. DNA fragments corresponding to the precursors of *BnMIR394a* and *BnMIR394b* were fused to 35S promoter and transformed into *Brassica napus*. Five transgenic independent lines were successfully obtained. The relative abundance of precursor miR394a/b (pre-miR394a/b) was further analyzed using RT-PCR, showing a similar increase in the transgenic lines (Fig 2A). In contrast, the expression of *BnLCR* in *35S*::*MIR394a/b* lines was very lower and its expression level was only 20–40% of the WT.

We further generated a cleavage-resistant version (*35S*::*Bnm6LCR*) by introducing six silent mutations in the miR394 binding site but without changing the protein sequences (Fig 2B). As a control, a vector carrying the intact *BnLCR* mRNA sequence was constructed. Comparative analysis by RT-PCR showed that the level of *BnLCR* mRNA in the *35S*::*Bnm6LCR* lines was 9.3–24.5 folds higher than that of WT, and its level in *35S*::*BnLCR* plants was increased only by 2.0–2.6 folds compared to WT (Fig 2C).

**Fig 3:**
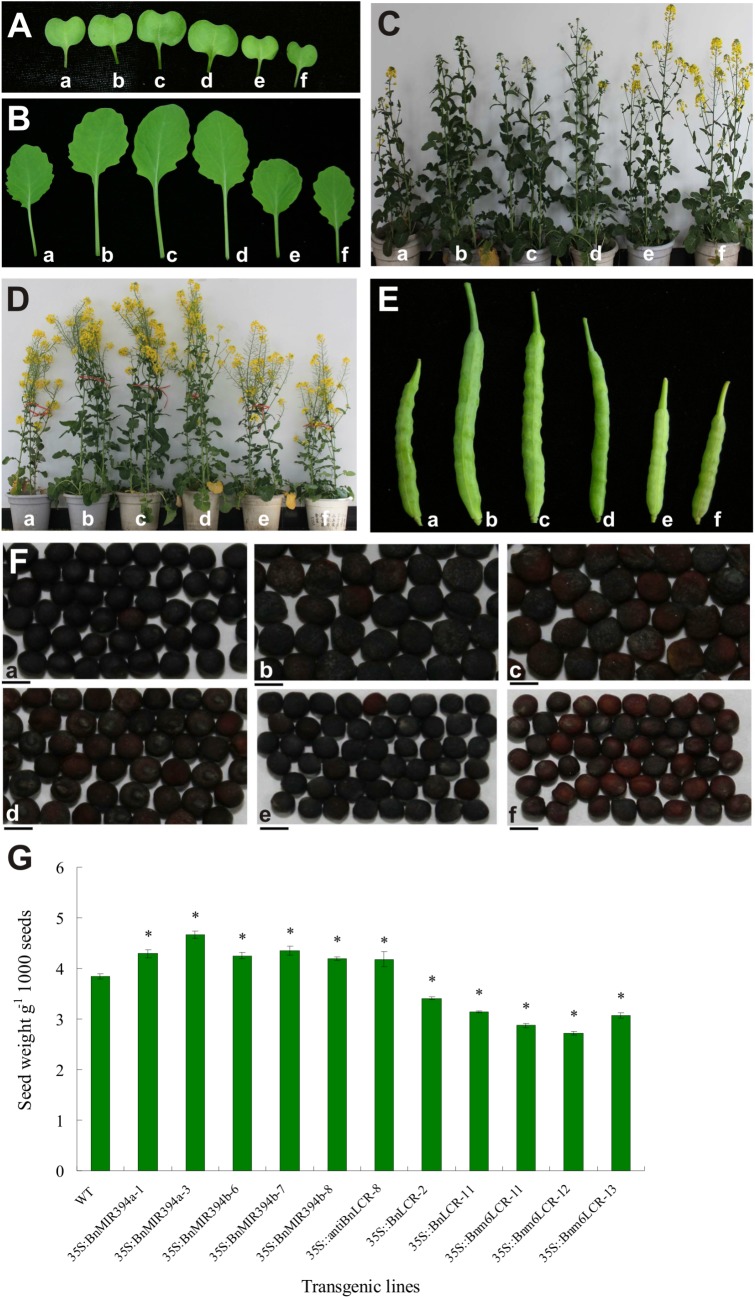
Phenotypes in various transgenic plants including (a) WT; (b) *35S*::*BnMIR394a*; (c) *35S*::*BnMIR394b*; (d) *35S*::*antiBnLCR* (transgenic plant over-expressing antisense mRNA of *BnLCR*); (e) *35S*::*BnLCR*; (f) *35S*::*Bnm6LCR*. (A, B) Cotyledon and leaf phenotypes of seven-day-old seedlings. (C) Flowering stage. (D) Plant height phenotype of the transgenic plants at flowering stage. (E) Phenotypes of pods. (F) Seed shapes of the transgenic plants. The scale bar in the graph indicates 5 mm. (G) Seed weight of different transgenic *Brassica napus* plants. Vertical bars represent SD of the mean. Asterisks indicate that mean values are significantly different between the transgenic plants and wild-type (WT) (*P*<0.05).

miR394 over-expressing *Brassica napus* plants showed a larger cotyledon and true leaf area, while the blade area was smaller in *35S*:*Bnm6LCR* plants compared to WT plants (Fig 3A and 3B). Interestingly, the *35S*::*BnMIR394a* and *35S*::*BnMIR394b* plants showed a lagged flowering time, whereas *35S*::*BnLCR* and *35S*::*Bnm6LCR* plants flowered earlier relative to WT (Fig 3C; Table 1). Moreover, miR394 over-expressing plants showed an enlarged size of plants, whereas *BnLCR* over-expressing plants had a shorten size compared to WT. We further made a comparative analysis of fruits between miR394 transgenic plants and WT, and found that although *35S*::*MIR394*, *35S*::*Bnm6LCR* and WT plants had bumpy appearance of pods, the *35S*::*MIR394* plants developed larger pods and the *35S*::*Bnm6LCR* plants showed reduced pods relative to WT (Fig 3E; Table 1). Consistent with the observation, the seed weight and size were enlarged in *35S*::*MIR394* plants, while the opposite phenotypes were found in *BnLCR* over-expressing plants (Fig 3F and 3G).

**Table 1:** Over-expression of miR394 modified phenotypes of *Brassica napus*.

Transgenic plants	Days after flowering	Leaves number	Height (cm)	Branch position (cm)	Diameter (cm)	Length of pods (cm)	Width of Pods (cm)
WT	0	17.22±0.38c	108.29±4.00b	37.60±2.74b	1.08±0.02e	8.32±0.07b	0.53±0.01b
*35S*::*BnMIR394a-1*	+4	22.44±0.51a	129.01±2.08a	62.43±3.62a	1.35±0.06a	11.03±0.83a	0.52±0.02b
*35S*::*BnMIR394a-3*	+3	20.67±0.33b	131.22±4.04a	62.75±1.77a	1.27±0.11b	10.98±0.49a	0.55±0.04b
*35S*::*BnMIR394b-6*	+2	20.67±0.58b	131.38±12.12a	60.85±4.01a	1.35±0.04a	10.06±0.57a	0.65±0.02a
*35S*::*BnMIR394b-7*	+3	19.56±0.69b	132.01±8.08a	62.68±3.42a	1.23±0.05c	8.97±0.27a	0.67±0.04a
*35S*::*BnMIR394b-8*	+2	19.22±0.51b	124.86±9.17a	58.63±0.53a	1.19±0.04d	10.12±0.69a	0.63±0.03a
Average	-2.8	20.51±0.52	129.70±7.10	61.47±2.67	1.28±0.06	10.23±0.57	0.61±0.03
*35S*::*Bnm6LCR-11*	-4	13.33±0.33d	100.17±3.05c	22.25±1.54c	0.98±0.02f	7.03±0.76c	0.49±0.03b
*35S*::*Bnm6LCR-12*	-4	14.01±0.67d	98.44±3.61c	23.03±2.85c	1.07±0.07e	6.21±0.17c	0.51±0.03b
*35S*::*Bnm6LCR-13*	-4	13.89±0.38d	94.00±3.01c	29.80±2.23c	0.94±0.07f	6.67±0.26c	0.50±0.03b
Average	-4	13.74±0.46	97.54±3.22	25.03±2.21	0.99±0.05	6.64±0.40	0.50±0.03

For each treatment, 30 plants were calculated. Values are the means ± SD. The different letters indicate that mean values are significantly different between the transgenic plants and wild-type (WT) (*P*<0.05). Branch position: the height where the first branch stem occurred.

**Fig 4:**
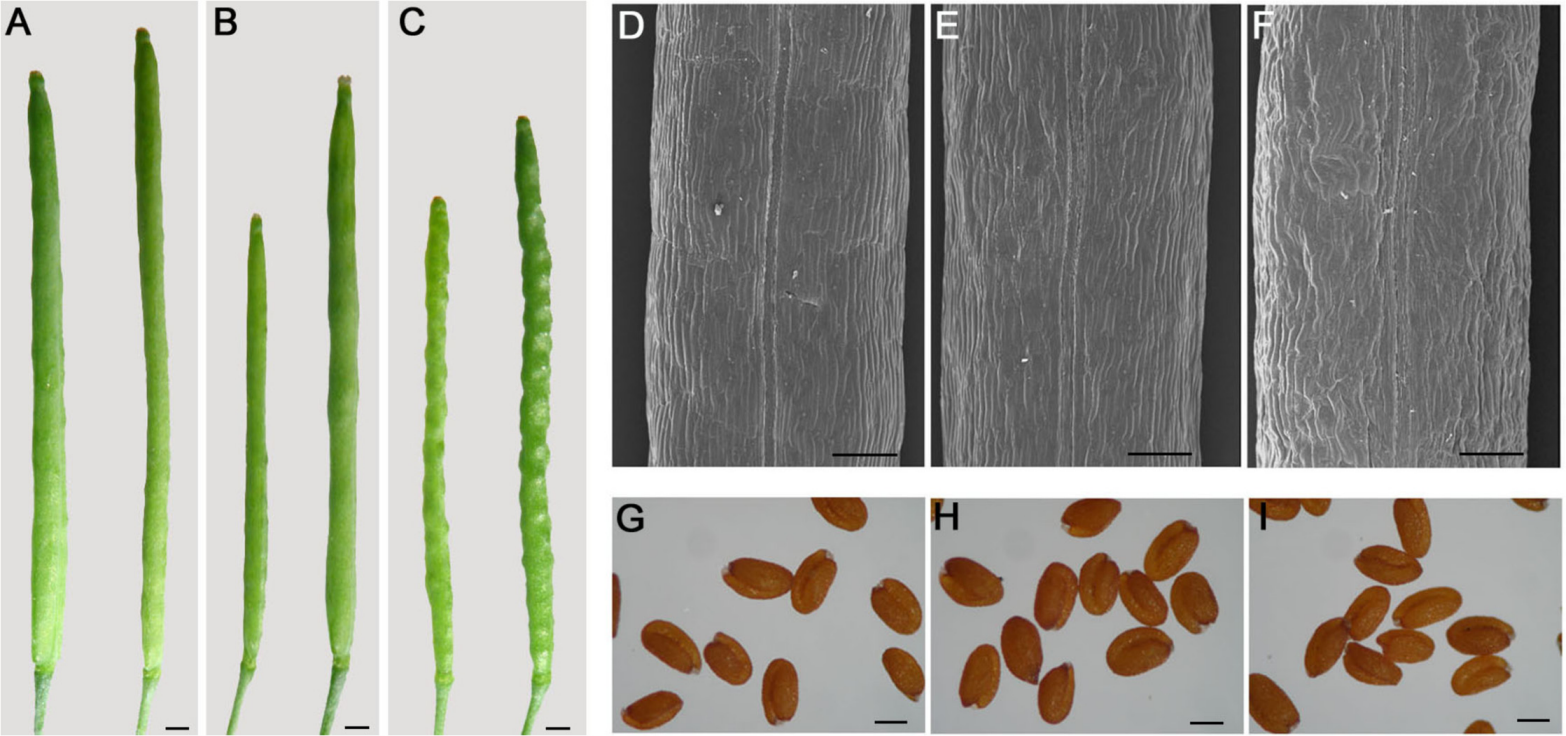
Fruit and seed development in transgenic Arabidopsis plants. A-C: Morphology of wild-type (A), *35S*::*MIR394a* (B) and *35S*::*m5LCR* (C) plant siliques. D-F: Close-up view of images showing the valve and replum tissues of Arabidopsis fruit (stage 17b) of the wild type (D), *35S*::*MIR394a* (E), and *35S*::*m5LCR* (F) plant siliques by a scanning electron microscope (SEM). G-I: The seed phenotypes of wild-type (G), *35S*::*MIR394a* (H) and *35S*::*m5LCR* (I) plants. Bars, 1 mm (A, B, C); 200μm (D, E, F); 300μm (G, H, I).

Recently, the developing siliques and seeds of Arabidopsis as a model system have been investigated [2, 4, 28]. The *35S*::*miR394* and *35S*::*m5LCR* (a miR394 cleavage-resistance version) transgenic Arabidopsis was used to examine the phenotypes of fruits and seeds. The silique wall of *35S*::*m5LCR* Arabidopsis was strongly wavy compared to wild-type, and the shape of individual seeds was apparent from the outside of the silique; but no apparent defects were detected in *35S*::*MIR394a* plant siliques (Fig 4A–4F). However, there was no difference of seed morphology between the transgenic plants and WT detected (Fig 4G–4I). We further quantitatively compared the silique width, length and seed number between *35S*::*MIR394a* or *35S*::*m5LCR Brassica napus* plants and wild type. Both *35S*::*MIR394a* and *35S*::*m5LCR* plants had a reduced silique width and length as well as the lower number of seeds compared to WT; however, only *35S*::*m5LCR* plants showed a significant decrease in silique length (Table 1).

### miR394 over-expression altered oil content and FA accumulation in *Brassica napus* seeds

Because miR394 over-expression altered the fruit and seed morphology of *Brassica napus*, it wa assumed that the oil accumulation and FA composition might be changed. Compared to wild-type, the total oil content significantly decreased in the seeds of *35S*::*BnMIR394a/b* plants, and the average oil contents in the *35S*::*BnMIM394a/b* seeds were only 79.56% of the wild-type (Table 2). The oil content was also reduced in *35S*:*m6BnLCR* transgenic plants, although the decrease of oil content was moderate as compared to wild-type.

**Table 2:** Total oil content of *Brassica napus* transgenic seeds.

*Transgenic plants*	*Total oil content (% dry weight)*	Percentage of the wild-type (%)
WT	41.45±0.51a	100
*35S*::*BnMIR394a-1*	30.39±0.05j	73.31
*35S*::*BnMIR394a-3*	33.18±0.18h	80.05
*35S*::*BnMIR394b-6*	32.09±0.28i	77.42
*35S*::*BnMIR394b-7*	34.95±0.17g	84.32
*35S*::*BnMIR394b-8*	34.27±0.42g	82.68
Average	32.98±0.22	79.56
*35S*::*Bnm6LCR-11*	38.08±0.22b	91.87
*35S*::*Bnm6LCR-12*	38.59±0.24b	93.10
*35S*::*Bnm6LCR-13*	36.41±0.03e	87.84
Average	37.69±0.16	90.93

Values are the means ± SD. The different letters indicate that mean values are significantly different between the transgenic plants and wild-type (WT) (*P*<0.05).

The major fatty acid species in the miR394 transgenic plants were analyzed by gas chromatography-mass spectrometry (GC-MS). Most of fatty acid species in the transgenic lines showed variations of percentage (Table 3). In *35S*::*MIR394a/b* plants, the average levels of C16:0 and C18:0 were increased approximately by 12.1 and 4.8%, respectively, as compared to the wild-type. C18:2 was increased by 13.6%, whereas C18:3 levels were reduced by about 19.1%. The very long-chain unsaturated fatty acid C22:1 species had a higher level (87.5%) in the *35S*::*MIR394a/b* transgenic lines, and the *35S*::*m6LCR* plants showed a changed level of the fatty acid species very similar to that of *35S*::*MIR394a/b*.

**Table 3:** Fatty acid (FA) compositions in *Brassica napus* transgenic and wild-type (WT) seeds (mol %).

Genotype	C16:0	C18:0	C18:1	C18:2	C18:3	C20:1	C22:1
WT	4.45±0.04e	2.28±0.01e	59.50±0.42c	18.05±0.17d	9.09±0.10a	0.78±0.01c	0.16±0.04e
*35S*::*BnMIR394a-1*	4.91±0.08b	2.89±0.05b	63.07±0.35a	17.52±0.15e	7.38±0.01c	0.71±0.01c	0.21±0.05d
*35S*::*BnMIR394a-3*	4.94±0.04b	2.17±0.06f	63.08±0.52a	17.07±0.14e	6.49±0.05d	0.76±0.04c	0.38±0.08a
*35S*::*BnMIR394b-6*	5.21±0.04a	2.44±0.20e	56.82±0.14c	23.55±0.28a	7.76±0.28b	0.83±0.06a	0.19±0.10e
*35S*::*BnMIR394b-7*	5.01±0.03b	2.28±0.03e	56.67±0.14c	21.97±0.26b	7.33±0.28c	0.88±0.13a	0.33±0.01b
*35S*::*BnMIR394b-8*	4.86±0.08b	2.15±0.01f	55.91±0.36d	22.37±0.18a	7.78±0.03b	0.79±0.01b	0.38±0.01a
Average	4.99±0.05	2.39±0.07	59.11±0.30	20.50±0.20	7.35±0.13	0.79±0.05	0.30±0.05
*35S*::*Bnm6LCR-11*	4.66±0.01c	3.06±0.01a	59.29±0.12c	19.67±0.01c	7.96±0.01b	0.70±0.01e	0.22±0.01d
*35S*::*Bnm6LCR-12*	4.77±0.03b	2.44±0.04e	61.75±0.43b	17.75±0.11e	7.03±0.06c	0.77±0.01c	0.30±0.02c
*35S*::*Bnm6LCR-13*	4.73±0.08b	2.56±0.11d	56.93±1.85c	19.99±0.57c	8.00±0.27b	0.69±0.01e	0.45±0.16a
Average	4.72±0.04	2.69±0.05	59.32±0.80	19.14±0.23	7.66±0.11	0.72±0.01	0.32±0.06

Mature seeds were used for the analysis by GC-MS. Values are the means ± SD. The different letters indicate that mean values are significantly different between the transgenic plants and wild-type (WT) (*P*<0.05).

### miR394 over-expression altered protein and GLS accumulation in *Brassica napus* seeds

Protein is one of the important constituents in *Brassica napus* seeds. Examination of the total protein content revealed that miR394 over-expressing plants accumulated more proteins than the wild-type, but no significant difference was observed between *LCR* over-expressing plants and wild-type (Table 4). GLS is a group of sulfur/nitrogen-containing secondary metabolites existing in many pungent plants such as mustard, rapeseed and horseradish. The natural chemicals derived from glucose and amino acids contribute to plant defense against pests and diseases [29]. Compared to wild-type, miR394 over-expressing plants usually had a relatively higher level of GLS, except for *35S*::*BnMIR394a-1* line (Table 4). In contrast, a generally lower level of GLS was determined in *35S*::*m6BnLCR* plants.

**Table 4:** Protein and GLS content in *Brassica napus* transgenic seeds.

*Genotype*	*Protein Content (% dry weight)*	*Percentage of wild-type (%)*	*GLS content (μmol dry weight)*	Percentage of wild-type (%)
WT	22.86±1.48b	100	52.77±3.30c	100
*35S*::*BnMIR394a-1*	25.33±0.94a	110.81	50.18±18.93d	95.09
*35S*::*BnMIR394a-3*	26.66±0.39a	116.62	76.96±13.58a	145.84
*35S*::*BnMIR394b-6*	26.11±0.42a	114.22	57.75±2.29b	109.43
*35S*::*BnMIR394b-7*	26.71± 0.14a	116.84	60.99±5.29ab	115.58
*35S*::*BnMIR394b-8*	26.51± 0.28a	115.97	68.47±7.58a	129.75
Average	26.26±0.43	114.89	62.87±9.53	119.14
*35S*::*Bnm6LCR-11*	21.65±0.12b	94.71	43.96±2.06e	83.30
*35S*::*Bnm6LCR-12*	21.48±0.44b	93.96	46.87±1.21d	88.82
*35S*::*Bnm6LCR-13*	22.11±0.33b	96.72	45.16±1.46de	85.58
Average	21.75±0.30	95.13	45.33±1.58	85.90

Values are the means ± SD. The different letters indicate that mean values are significantly different between the transgenic plants and wild-type (WT) (*P*<0.05).

### Regulation of seed lipid synthesis-responsive genes by over-expression of miR394

**Fig 5:**
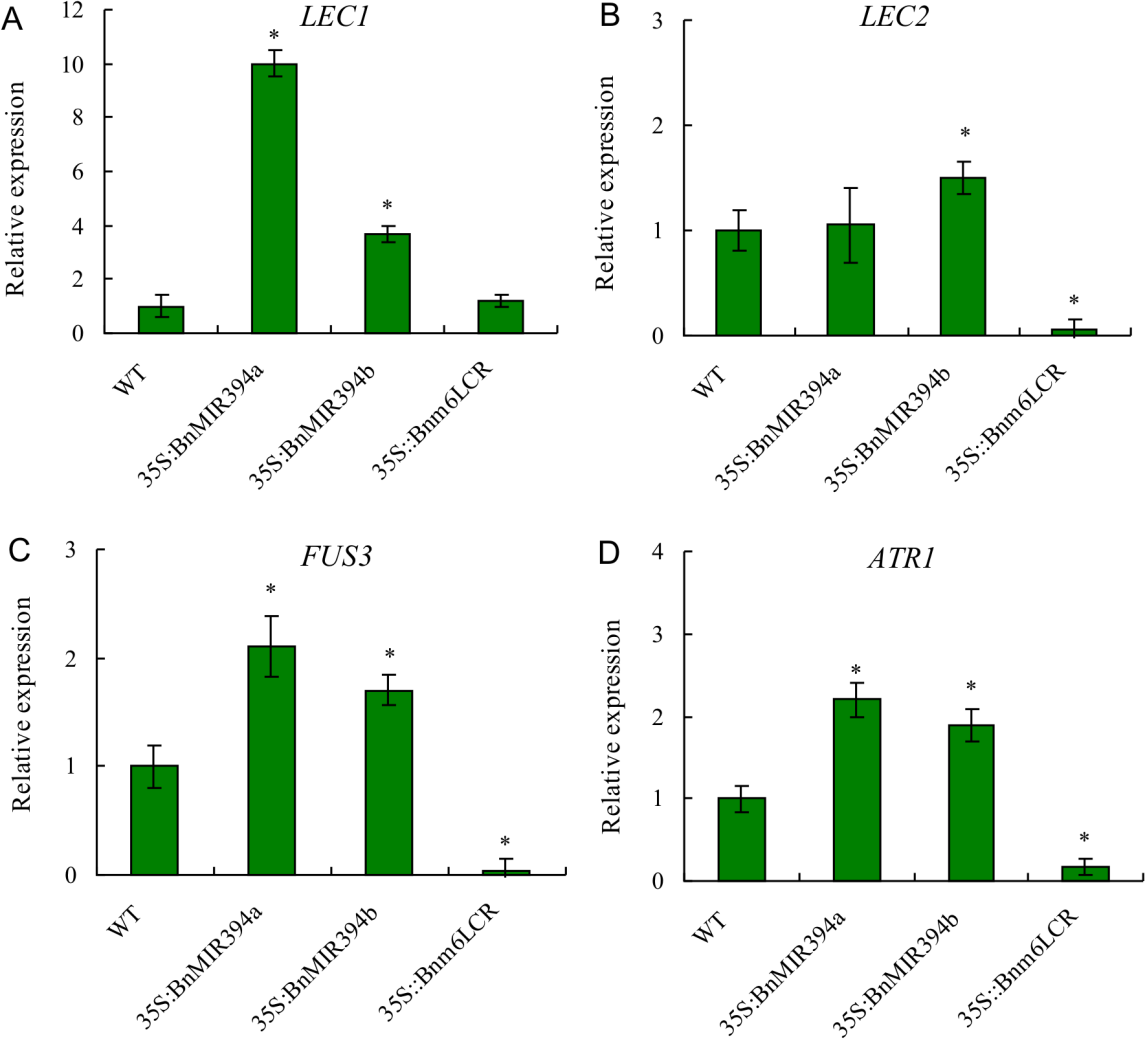
Real-time PCR analysis of *Brassica napus* genes *LEAFY COTYLEDON1* (*LEC1*), *LEAFY COTYLEDON2* (*LEC2*), *FUSCA3* (*FUS3*) involved in oil synthesis regulation and *ALTERED TRYPTOPHAN REGULATION1* (*ATR1*) involved in glucosinolate synthesis. The expression levels were measured in developing seeds 30 days after pollination (DAP). The relative expression level of each gene was normalized using BnACTIN2 as an internal control. Vertical bars represent the standard deviation of the mean treatments (*n* = 3). Asterisks indicate that mean values are significantly different between the *35S*:*MIR394* or *35S*:*m6LCR* plants and WT (*p* < 0.05).

To get an insight into the lipid biosynthetic mechanism mediated by miR394, four major genes *LEAFY COTYLEDON1* (*LEC1*), *LEAFY COTYLEDON2* (*LEC2*), *FUSCA3* (*FUS3*) involved in regulation of seed oil synthesis and *ALTERED TRYPTOPHAN REGULATION1* (*ATR1*) for seed GLS synthesis were analyzed. *LEC1* encodes an NFY-B-type or CCAAT-binding factor-type transcription factor [30]. *LEC2* [31] and *FUS3* [32] encode proteins also belong to the plant-specific B3 transcription factor gene family. The three key genes have been identified as controllers of seed maturation in Arabidopsis [33]. Compared to wild type, expression of *LEC1* in *35S*::*BnMIR394a/b* plants was increased by 10.0 and 3.7 fold, respectively, but expression of *LEC2* in *35S*::*BnMIR394b* plants was weak (Fig 5A and 5B). *FUS3* expressed moderately in *35S*::*BnMIR394a/b* plants and its transcripts were increased by 2.1 and 1.7 fold, respectively (Fig 5C). *ATR1*, a MYB transcription factor gene, controls GLS homeostasis by activating several enzymes responsible for biosynthesis of GLS. Expression of *ATR1* in *35S*::*BnMIR394a/b* plants showed a pattern similar to *LEC1* and *FUS3* (Fig 5D). In general, the *35S*::*Bnm6LCR* plants showed depressed expression of *LEC2*, *FUS3* and *ATR1* or no change in *LEC1* transcription.

### miR394-regulated fruit and seed development was possibly involved in auxin

**Fig 6:**
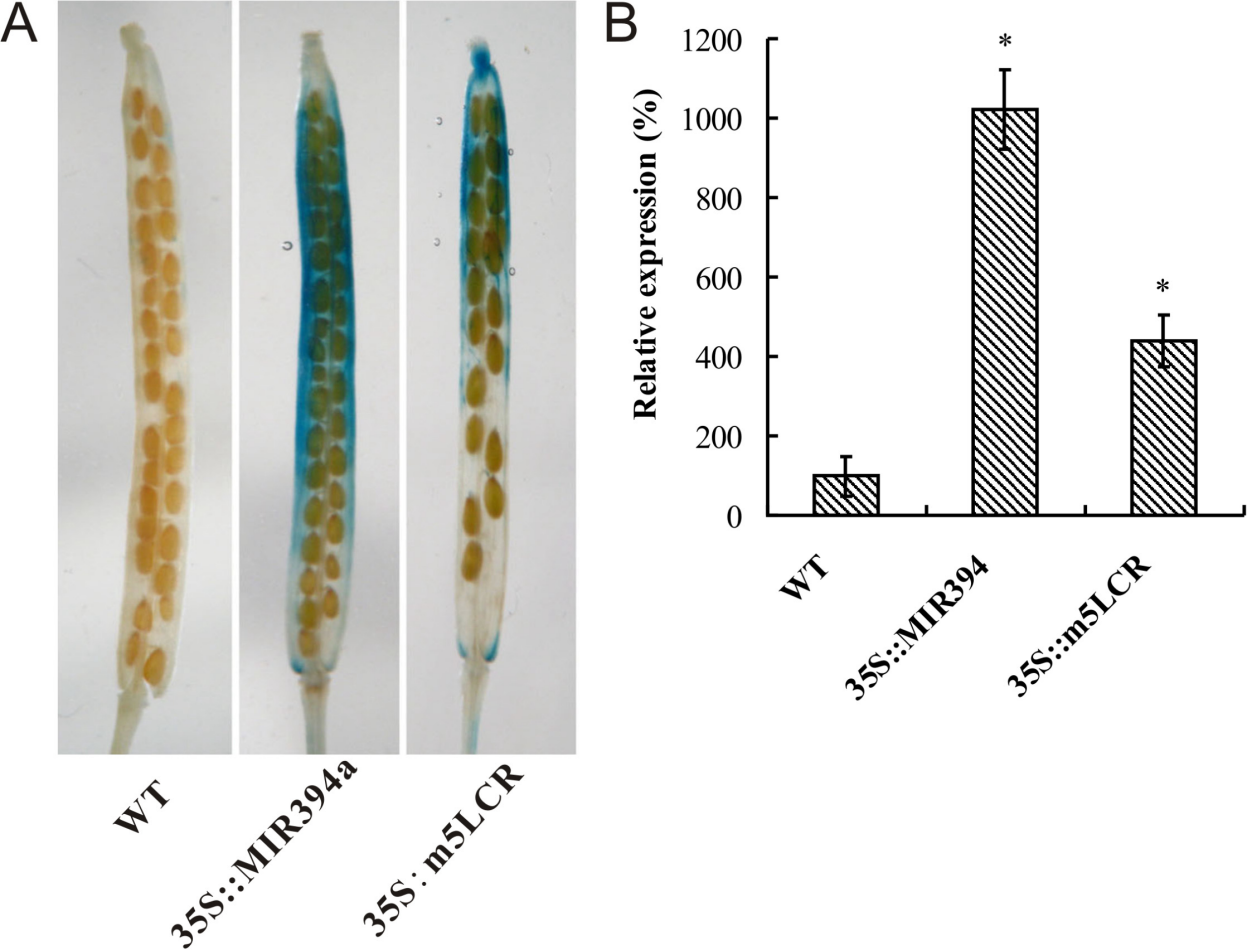
DR5-GUS expression in Arabidopsis wild type (Col), *35S*::*MIR394a* and *35S*::*m5LCR* fruits. A: Visualization of DR5-GUS reporter in stage 17 fruits of *pDR5*::*GUS/Col*, *pDR5*::*GUS/MIR394a* and *pDR5*::*GUS/m5LCR* transgenic plants. B: Relative quantification of GUS intensity in stage 17 fruits of wild type (Col), *35S*::*MIR394a* and *35S*::*m5LCR*. The intensity of GUS coloration was statistically quantified using Adobe Photoshop CS software. Twenty fruits were measured. The relative intensity of WT is considered as 100%. Vertical bars represent the standard deviation of the mean treatments (*n* = 3). Asterisks indicate that mean values are significantly different between the *35S*:*MIR394a* or *35S:m5LCR* plants and WT (*p* < 0.05).

Recent studies have shown that fruit and seed development is involved in local auxin abundance because it directs organ initiation and mediates tissue patterning [34]. To investigate whether miR394-regulated seed development was involved in auxin signal, transgenic plants with a *pDR5*::*GUS* reporter was constructed to visualize auxin responses in Arabidopsis fruits during the seed development. In wild-type developing fruits, the *pDR5*::*GUS* expression was always low and the GUS staining was nearly undetectable (Fig 6). In contrast, the *pDR5-GUS* expression of *35S*:*miR394* plant was significantly higher than that of wild-type. The *35S*::*m5LCR* fruits had moderate expression of GUS gene. The *DR5-GUS* expression mainly occurred along the valve. A statistic analysis was made on *DR5-GUS* staining intensity using the method of Adobe Photoshop CS software [35], which showed a similar result in the corresponding fruits.

## Discussion

Seeds of Brassicaceae family such as *Brassica napus* accumulate lipids mainly in the form of triacylglycerols as the major source of energy required for seed germination and post-germination growth. Although genetic screens and molecular identification have allowed for characterization of a number of genes related to seed development, oil production and biosynthesis of fatty acids in both Arabidopsis and *Brassica napus* [4, 5, 10, 36–38], investigations on miRNAs involved in the fruit and seed development with regard to oil metabolism are lacking. Our results have shown that miR394 was expressed in flower buds, with a low abundance at the latter stage of seed development; in contrast, expression of *LCR* was relatively higher during the seed maturation. These results imply that both miR394 and *LCR* are possibly involved in the fruit and seed development. Over-expression of miR394 resulted in pleiotropic phenotypes in seed morphology. Compared to wild-type, *35S*::*miR394* rapeseed exhibited larger pods and seed bodies (Fig 3). In addition, miR394 over-expression let to the change in the seed oil content and FA composition (Tables 1 and 2). The phenotypes regulated by several miRNAs have been recently reported [39, 40]. For example, miR160, targeting *AUXIN RESPONSE FACTOR10* (*ARF10*), *ARF16* and *ARF17*, plays important roles in seed germination and post-germination; transgenic plants expressing an miR160-resistant form of *ARF10* displayed multiple developmental defects such as serrated leaves, curled stems, contorted flowers and twisted siliques [40]. The multi-target genes of miR160 should be responsible for the pleiotropic phenotypes.

Seed oil is mainly synthesized during the seed maturation phase. Alteration of seed development may affect the oil production and FA composition. We analyzed oil accumulation in the transgenic plants and found that the oil content in miR394 over-expressing *Brassica napus* was reduced. This could be attributed to the enlarged seed body of *35S*::*miR394* plants that made the seed oil content “diluted”. The reduced oil content in *35S*::*miR394* plants might be the result of the increased proportion of seed protein and GLS and other seed storage compounds. In higher plants, oil production and FA synthesis is a highly coordinated process involved in not only FA synthetic metabolism but also carbon and amino acid flux in the cells [41]. It is considered that the seed oil content is negatively correlated with the seed protein content [42]. In this study, over-expression of *BnLCR* also reduced the oil production compared to wild-type, although the protein content was not significantly lowed in the *35S*::*m6BnLCR* plants. The complicated process for the repressed oil production in the transgenic plants remains to be elucidated.

The altered seed development in the *35S*::*miR394* rapeseed was biologically linked to the changed FA composition. Over-expression of miR394 led to the high proportion of C16:0 and C18:0, two end products of condensation reactions in the storage oil, and more significant abundance of unsaturated C22:1 species was found in the seeds. Conversely, the unsaturated C18:3 species was lower in miR394 over-expressing plants. Regulation of FA biosynthesis has been proposed to act at multiple levels, of which transcriptional control has been considered as a major molecular process [33]. To help understand the regulatory mechanism in miR394 over-expression plants, we analyzed the expression of three master regulatory genes coding for LEC1, LEC2, and FUS3 involved in FA synthesis and seed development, [31–33]. Both *LEC1* and *LEC2* encode B3 transcription factors, which positively regulate oil synthesis but are not to function in a liner pathway [32, 33, 37]. It was reported that over-expression of *LEC1* in Arabidopsis promoted the FA accumulation through a global induction of many FA biosynthetic genes [33]. In this study, expression of *LEC1* was up-regulated in the *35S*::*miR394* plants. However, the expression of *LEC2* in the *35S*::*miR394* plants was low. In agreement with *LEC1*, expression of *FUS3* was positively but moderately regulated in *35S*::*miR394a* plants. Brassicaceae is one of the few plant families that are able to produce GLS which tends to accumulate in developing seeds [43]. GLS accumulation was elevated in the miR394 over-expressing plants. Consistent with the observation, the transcript level of *ATR1* responsible for biosynthesis of GLS was induced in *35S*::*miR394* plants. These results suggest that miR394-regulated GLS accumulation was mediated by *ATR1*. GLS are a class of secondary metabolites and regarded as defense compounds [43]. The elevated level of GLS in *35S*::*miR394* plants suggests that the plants may have potentials to work against biotic and abiotic stresses through GLS. Identification of the correlation of miR394 and GLS will help to understand the role of miR394 played in plant responses to stress such as pathogen attack and its resistant mechanisms.

Seed development (*e*.*g*. size) in higher plants is regulated by a number of intracellular components. Several factors (such as Auxin response factor 2 and Apetala 2) that act as a regulator of seed size have been identified [44]. Li et al. (2008) identified an ubiquitin receptor DA1 (Large) that acts synergistically with the E3 ubiquitin ligase ENHANCER1 OF DA1 (EOD1)/BIG BROTHER to regulate the final size of seeds in *Arabidopsis thaliana* [45]. Recently, a RING-type protein with E3 ubiquitin ligase activity encoded by *DA2* has been characterized, and this protein regulates seed size by restricting cell proliferation in the maternal integuments of developing seeds [44]. These results indicate that E3 ubiquitin ligases play a role in seed development. With regard to the plant hormones, early genetic and molecular studies demonstrated that the SCF^TIR1^ ubiquitin ligase is able to positively regulate the auxin signaling in Arabidopsis [46]. The SCF^TIR1^ complex mediates auxin response by targeting members of the auxin/indoleacetic acid (Aux/IAA) family of transcriptional regulators for ubiquitin-mediated proteolysis in response to an auxin stimulus [47]. Because the phytohormone auxin is involved in many aspects of cellular and developmental responses in plants including fruit initiation and seed development [48], manipulation of the SCF^TIR1^ ubiquitin ligase would change the ubiquitin-mediated regulation of auxin signaling during seed development. However, as there are very limited numbers of signaling components identified from the auxin pathway, the molecular mechanisms for the ubiquitin-mediated regulation of auxin signaling during seed development are largely unknown [44, 49]. The present studies provided evidence that the *DR5-GUS* was strongly expressed in the fruit of both *35S*:*miR394* and *35S*:*m5LCR* transgenic plants. This result is very similar to our previous report that DR5-GUS was also abnormally accumulated in the leaf tips or hydathodes of Arabidopsis [20]. Furthermore, expression of several auxin responsive genes such as IAA3, IAMT1, PIN1, PIN3, PIN4, and PIN7 has been upregulated in *35S*:*miR394* plants [20]. These results suggest that auxin in these organs was most likely to be involved in the process. The auxin profile has been characterized during the development of pod wall, dehiscence zone and seeds of *Brassica napus* pods [50]. A decrease in auxin content prior to moisture loss in the pods of *Brassica napus* was specifically detected in the dehiscence zone, and a minimized auxin was required for seed dispersal in Arabidopsis [34]. Consistent to the reports, a very low level of DR5-GUS was detected in the siliques of wild-type Arabidopsis. The high level of DR5-GUS detected in the siliques of miR394 over-expressing plants suggests that auxin distribution in these tissues was impaired due to the over-expression of miR394.

In conclusion, the present study identified miR394 and its target gene *LCR* from *Brassica napus*, which plays a role in mediating seed development with regard to seed morphology and composition of storage compounds. The coordinate expression of miR394 and *LCR* is necessary for proper fruit development and seed formation. This observation allowed us to postulate that *LCR* as an F-box protein gene may be involved in the mechanism responsible for regulating the abundance of putative components via protein degradation by the ubiquitin-dependant proteosome. Further investigation on miR394 and *LCR* with interaction to components in the auxin-responsive pathway will help understand how miR394 and *LCR* regulates its downstream genes responsible for the altered seed development.

## Supporting Information

S1 File Contains Figure A, miR394-guided cleavage site on *BnLCR*. Figure B, CDS and deduced amino acid sequences of *BnLCR*. Figure C, Phylogenetic relationships of LCR between *Brassica naups* and other plant species. The name and GenBank accession number as follow: *Ricinus communis* (XP_002514903.1), *Populus trichocarpa* (XP_002297845.1), *Citrus trifolita* (ACL51019.1), *Prunus persica* (XP_007225618.1), *Malus x domestica* (XP_008353048.1), *Vitis vinifera* (XP_010655984.1), *Cucumis sativus* (XP_004140594.1), *Glycine max* (XP_006591447.1), *Medicago truncatula* (XP_003601765.1) *Brassica napu* (CDX84930.1), *Arabidopsis thliana* (NP_564278.1), *Sorghum bicolor* (XP_002459002.1), *Zea mays* (XP_008656925.1), *Oryza sativa* Japonica Group (NP_001045243.2), *Brachypodium distachyon* (XP_003564961.1), *Triticum aestivum* (AEK78079.1), and *Hordeum vulgare* (BAJ95420.1). Figure D, Genomic organization of *Brassica napus* LCR gene. The open boxes and lines denote the coding regions and introns, respectively. The gray box denotes the positions of the putative transit peptide sequences. The nucleotide-sequence length of each domain was marked by corresponding numbers. Table A, Basic physicochemical properties of the yellow-brown soil (Eutric gleysols) used for *Brassica napus* cultivation. Table B, Primers used in this study.
(DOC)
