# Supplementary material for: Altered Fruit and Seed Development of Transgenic Rapeseed (Brassica napus) Over-Expressing MicroRNA394
Source: PLoS One. 2015 May 15;10(5):e0125427. doi: 10.1371/journal.pone.0125427 (PMC4433277; doi:10.1371/journal.pone.0125427)
Supplement: S1 File — Figure B, CDS and deduced amino acid sequences of BnLCR. Figure C, Phylogenetic relationships of LCR between Brassica naups and other plant species. The name and GenBank accession number as follow: Ricinus communis (XP_002514903.1), Populus trichocarpa (XP_002297845.1), Citrus trifolita (ACL51019.1), Prunus persica (XP_007225618.1), Malus x domestica (XP_008353048.1), Vitis vinifera (XP_010655984.1), Cucumis sativus (XP_004140594.1), Glycine max (XP_006591447.1), Medicago truncatula (XP_003601765.1) Brassica napu (CDX84930.1), Arabidopsis thliana (NP_564278.1), Sorghum bicolor (XP_002459002.1), Zea mays (XP_008656925.1), Oryza sativa Japonica Group (NP_001045243.2), Brachypodium distachyon (XP_003564961.1), Triticum aestivum (AEK78079.1), and Hordeum vulgare (BAJ95420.1). Figure D, Genomic organization of Brassica napus LCR gene. The open boxes and lines denote the coding regions and introns, respectively. The gray box denotes the positions of the putative transit peptide sequences. The nucleotide-sequence length of each domain was marked by corresponding numbers. Table A, Basic physicochemical properties of the yellow-brown soil (Eutric gleysols) used for Brassica napus cultivation. Table B, Primers used in this study. (DOC) [file pone.0125427.s001.doc]

**Title of article**: Altered fruit and seed development of transgenic rapeseed (*Brassica napus*) over-expressing microRNA394

**Full names of all authors:** Jian Bo Song, Xia Xia Shu, Qi Shen, Bo Wen Li, Jun Song, Zhi Min Yang

**Table A** Basic physicochemical properties of the yellow-brown soil (Eutric gleysols) used for *B. napus* cultivation.

| **Soil type** | **pH** | **Organic carbon (%)** | **Texture** | | | **CEC**  **(cmol kg^-1^)** | **Available**  **K (mg kg^-1^)** | **Available**  **P (mg kg^-1^)** | **Total N (%)** |
| --- | --- | --- | --- | --- | --- | --- | --- | --- | --- |
|  |  |  | Clay(%) | Silt(%) | Sand(%) |  |  |  |  |
| Yellow-brown soil | 7.24 | 0.6612 | 25.11 | 39.58 | 35.31 | 23.47 | 101.3 | 33.9 | 0.158 |

**Table B** Primers used in this study.

| Genes | Primers used for gene cloned |
| --- | --- |
| BnLCR | Forward: 5'-ATGGAAGAAGAGCTTGCCATG-3'  Reverse: 5'-TCAAGCCCTAGCCGTAGGGG-3' |
| Bnm6LCR | a: 5′- ACTAGTATGGAAGAAGAGCTTGCCATG -3′  b: 5′-CGTAAAACTCCAAGCACCATATGTTGGGCATCCTATCGACTTCTT-3′  c: 5′-AAGAAGTCGATAGGATGCCCAACATATGGTGCTTGGAGTTTTACG-3′  d: 5′-GGACTAGTTCAAGCCCTAGCTGTAGGGG-3′ |
| BnMIR394a | Forward: 5'-CCATGGATACAACTTCATGAGCGGG-3'  Reverse: 5'-ACTAGTGCTCACCAGATTCAAAACA-3' |
| BnMIR394b | Forward: 5'-CCATGGCGACAGAAAGGAACGAGT-3'  Reverse: 5'-ACTAGTACGAGCCAATGCCTTATC-3' |
| AtMIR394a | 5'-CATGCCATGGCCGTCATAAAGAGAACTCATCTGCC-3′  5'-GACTAGTTTCATCGCCAAGAAACAAATC-3′ |
| AtMIR394b | 5'-CATGCCATGGCATGCGGGTTTGACAAAAG-3′  5'-GACTAGTCAAGTAACGCTCACAGTCAC-3′ |
| AtMIR394aP | 5'-CGGAATTCTGGGCAGACGATATTACT-3′  5'-GGACTAGTAAAATCTTTGTCAAACCCT-3′ |
| AtMIR394bP | 5'-CGGAATTCTTCACTATGCGACAAAC-3′  5'-GGACTAGTAGACTCTTTTGTCAAACCC-3′ |
| AtLCR | 5′- GAAGATCTATGGAAGAAGAGCTTGCCATG -3′  5′- GACTAGTATGGAAGAAGAGCTTGCCATG -3′ |
| Atm5LCR | a: 5′- GAAGATCTATGGAAGAAGAGCTTGCCATG -3′  b: 5′- GCACCATATGTTCGGCATGCGATCAACTTCCTTCCACAACAGTGT -3′  c: 5′- ACACTGTTGTGGAAGGAAGTTGATCGCATGCCGAACATATGGTGC -3′  d: 5′- GACTAGTATGGAAGAAGAGCTTGCCATG -3′ |
| AtLCRP | 5'-CCGGAATTCTATTGTCTTGATACCAA  5'-GGACTAGTGCTTCATCTTTTTCCATGGT |
|  |  |
| Gene | Primer sequence (5'-3') for semi quantitative RT-PCR analysis |
| *BnLCR* | Forward : 5'-CCAGCGATGGCTACAAG-3'  Reverse : 5'-GCCTAGACAATTCACCCT-3' |
| pre-miR394a | Forward :5'-ATACAACTTCATGAGCGGG-3  Reverse :5'-GCTCACCAGATTCAAAACA-3 |
| pre-miR394b | Forward :5'-CGACAGAAAGGAACGAGT-3'  Reverse :5'-ACGAGCCAATGCCTTATC-3' |
| *BnActin* | Forward : 5'-TCCCGAGTATTGTTGGTC-3'  Reverse : 5'-CTGCTCTTAGCCGTCTCC-3' |
|  |  |
| Genes | Primers for real-time RT-PCR |
| BnLEC1 | Forward: 5'- TATCTTGCCGCAGCAACAACCAAG-3'  Reverse: 5'-TTCACCGGTCACGAAGCTGATGTA-3' |
| BnLEC2 | Forward: 5'-ACAAGAATCGCTCGCACTTCTCCA-3'  Reverse: 5'-AAGCATCCGATGAGTGAAGAGGCT-3' |
| BnFUS3 | Forward: 5'-TCCATCATCGTCCAGGGTTTGGAT-3'  Reverse: 5'-AAGCATCCGATGAGTGAAGAGGCT-3' |
| BnATR1 | Forward: 5'-TGGCCTTATGGAGGAGTTAGAGGA-3'  Reverse: 5'-ATCAGTTACGTCAACCTCAGGCGA-3' |
| BnACTIN | Forward: 5'-ATCAGTTACGTCAACCTCAGGCGA-3'  Reverse: 5'-CGTAGGCAAGCTTCTCTTTAATGTC-3' |


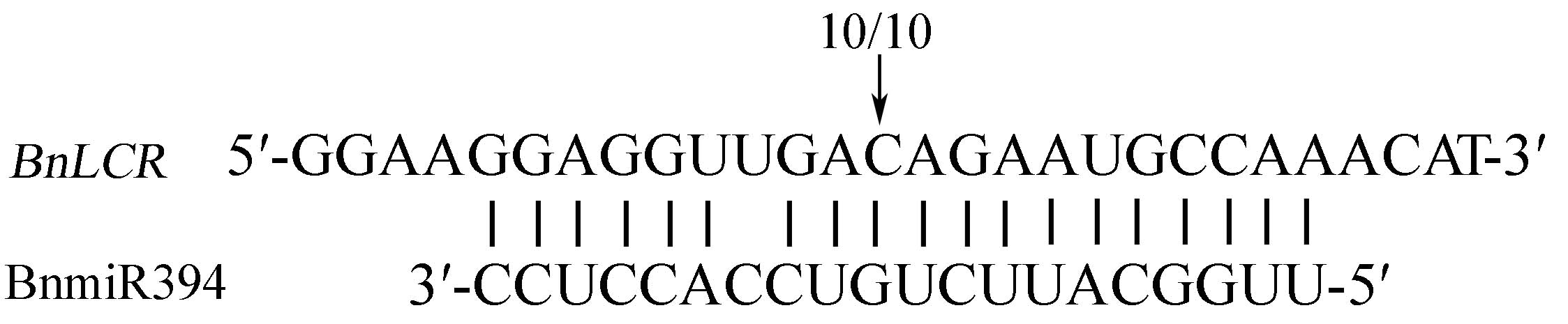


**Figure A.** miR394-guided cleavage site on *BnLCR*

**Figure B.** CDS and deduced amino acid sequences of *BnLCR*

CDS sequences of *BnLCR* (1404 bp)

atggaagaagagcttgccatgctcaaacagttcatcggccagcttcaagagctcttgcacaacggctctcatcctccttcttcacctccctcttcctcttcctcttcttcttcgtcgtcttttatagttctacacaaccctcactatcagaacggatggtgtttgccctttactgaggaaacttctgctgatgattcttgtgatcttctaatggctcctggaaagaggcctggggggatcttcaacatgttagagaccgtcaagcaacctgtcaaacgttctcgaaaagacaagaagaatcaaggaaaatcatccaccgaaggagatggaaacatggatcaagaaatctggcaggagtttcctcatgatctcttcgaatctgttgtctccagactccccatccctaagtttttccagttccgtgcagtttgtcgtaaatggaacgctctcatcgattcagacagcttctcccgctgctgcaccaacctccctcagaccatcccatggttctacaccataacccacgacaatgtcaactcgggacaagtctacgacccttcttccaagaaatggcaccatcccgttatccctgcacttcccaagaagactattgtcttgcctatggcatccgcgggaggtctagtgtgcttcctcgacattggccaccggaacttctacgtgagcaaccctctgaccaagtctttcagagagttgccagcgaggtcgttcaaggtgtggtctcgtgtcgcagtaggaatgactcttaacggaaactccaccagcgatggctacaaggtcttgtgggttggatgcgaaggagagtacgaagtttatgattcctcgagcaacgtatggaccaaacgagggaccatcccgtcatacataaagctccccgtactgctcaacttcaagtcgcagccggtggctatccaaagcacgctttacttcatgttaacggagcccgaagggatattgtcctacgacatggtctcagggcagtggaagcagtacatcataccgggtccaccggacctgagcgatcacacgctagcggagtgcggggagaggttgttgctggtgggtcttctgacgaaaaacgctgccacgtgcgtgtgcatatgggagctgcagaagatgacgctgctgtggaaggaggttgacagaatgccaaacatatggtgcttggagttttacggaaagcacgtgaggatgaattgtctaggcaacaaaggttgtctgatgatgttgtccttgaggtccagacagatgaaccgtctgattacctacaatgctgttactagggaatgggccaaggtccctggctgtaccgttcctcgtgggagaaaaaggctttggatcgcttgcggaacggcgtttcatccctcccctacagctagggcttga

Sequences of amino acids (467 aa)

MEEELAMLKQFIGQLQELLHNGSHPPSSPPSSSSSSSSSSFIVLHNPHYQNGWCLPFTEETSADDSCDLLMAPGKRPGGIFNMLETVKQPVKRSRKDKKNQGKSSTEGDGNMDQEIWQEFPHDLFESVVSRLPIPKFFQFRAVCRKWNALIDSDSFSRCCTNLPQTIPWFYTITHDNVNSGQVYDPSSKKWHHPVIPALPKKTIVLPMASAGGLVCFLDIGHRNFYVSNPLTKSFRELPARSFKVWSRVAVGMTLNGNSTSDGYKVLWVGCEGEYEVYDSSSNVWTKRGTIPSYIKLPVLLNFKSQPVAIQSTLYFMLTEPEGILSYDMVSGQWKQYIIPGPPDLSDHTLAECGERLLLVGLLTKNAATCVCIWELQKMTLLWKEVDRMPNIWCLEFYGKHVRMNCLGNKGCLMMLSLRSRQMNRLITYNAVTREWAKVPGCTVPRGRKRLWIACGTAFHPSPTARA


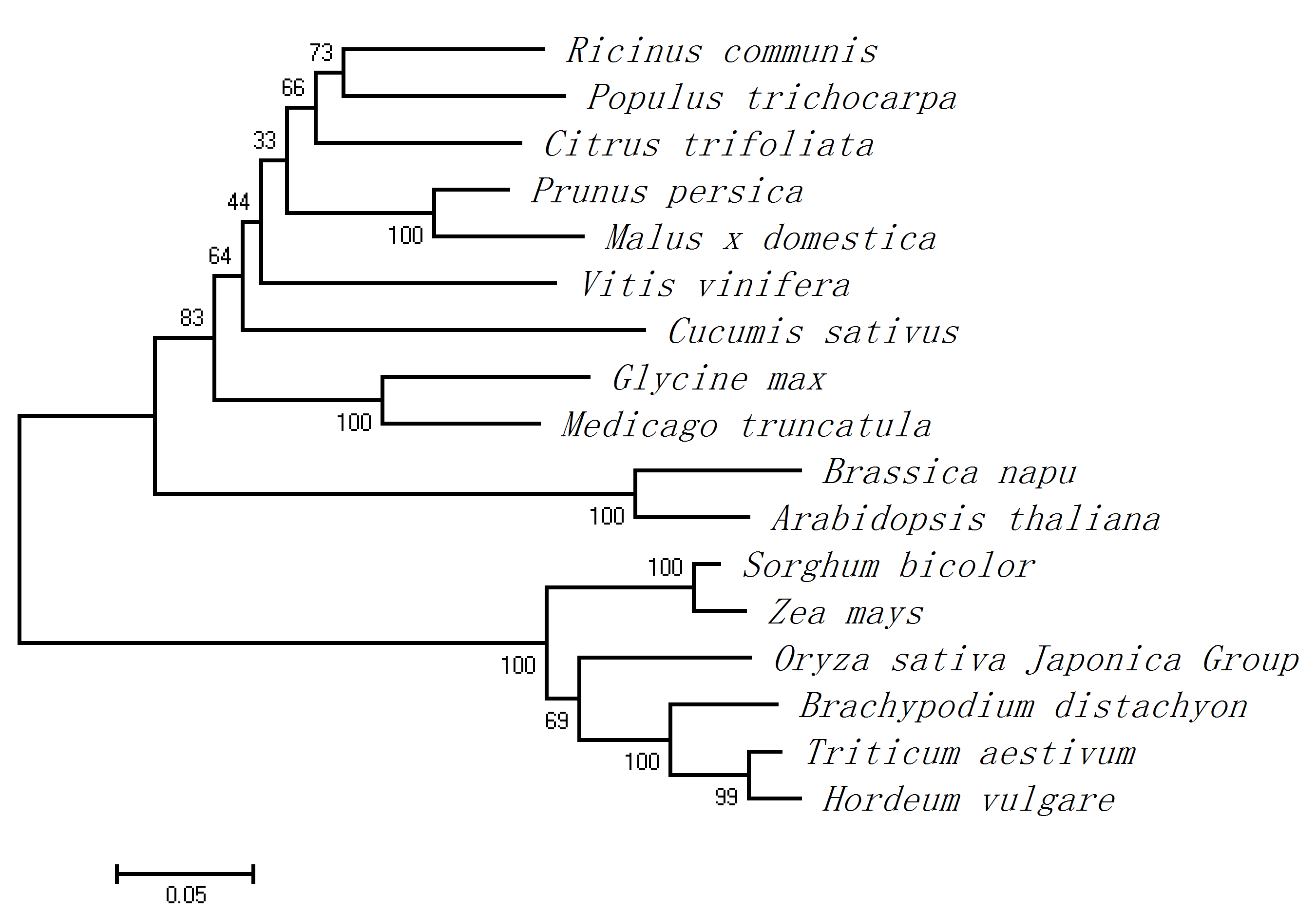


**Figure C.** Phylogenetic relationships of LCR between *Brassica naups* and other plant species. The name and GenBank accession number as follow: *Ricinus communis* (XP_002514903.1), *Populus trichocarpa* (XP_002297845.1), *Citrus trifolita* (ACL51019.1), *Prunus persica* (XP_007225618.1), *Malus x domestica* (XP_008353048.1), *Vitis vinifera* (XP_010655984.1), *Cucumis sativus* (XP_004140594.1), *Glycine max* (XP_006591447.1), *Medicago truncatula* (XP_003601765.1) *Brassica napu* (CDX84930.1), *Arabidopsis thliana* (NP_564278.1), *Sorghum bicolor* (XP_002459002.1), *Zea mays* (XP_008656925.1), *Oryza sativa* Japonica Group (NP_001045243.2), *Brachypodium distachyon* (XP_003564961.1), *Triticum aestivum* (AEK78079.1), and *Hordeum vulgare* (BAJ95420.1).


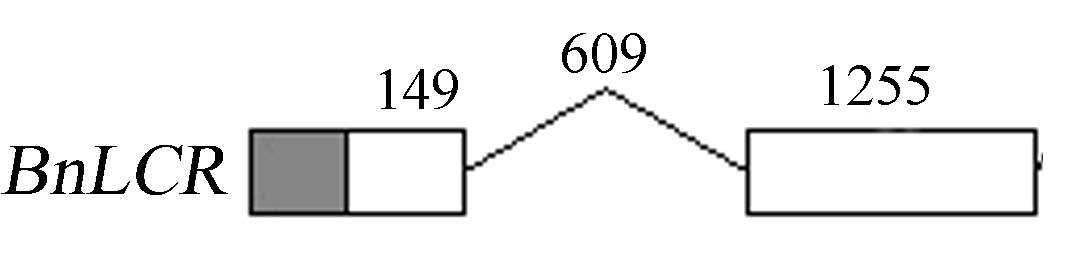


**Figure D.** Genomic organization of *Brassica napus* LCR gene. The open boxes and lines denote the coding regions and introns, respectively. The gray box denotes the positions of the putative transit peptide sequences. The nucleotide-sequence length of each domain was marked by corresponding numbers.
